# Supplementary material for: Engrailed‐1 Promotes Pancreatic Cancer Metastasis
Source: Adv Sci (Weinh). 2023 Dec 18;11(6):2308537. doi: 10.1002/advs.202308537 (PMC10853725; doi:10.1002/advs.202308537)
Supplement: Supplementary file 1 — Supporting Information [file ADVS-11-2308537-s001.pdf]

ADVANCED SCIENCE
Open Access

Supporting Information

for *Adv. Sci.*, DOI 10.1002/advs.202308537

Engrailed-1 Promotes Pancreatic Cancer Metastasis

Jihao Xu, Jae-Seok Roe, EunJung Lee, Claudia Tonelli, Keely Y. Ji, Omar W. Younis, Tim D.D. Somervile, Melissa Yao, Joseph P. Milazzo, Herve Tiriac, Anna M. Kolarzyk, Esak Lee, Jean L. Grem, Audrey J. Lazenby, James A. Grunkemeyer, Michael A. Hollingsworth, Paul M. Grandgenett, Alexander D. Borowsky, Youngkyu Park, Christopher R. Vakoc, David A. Tuveson and Chang-Il Hwang**

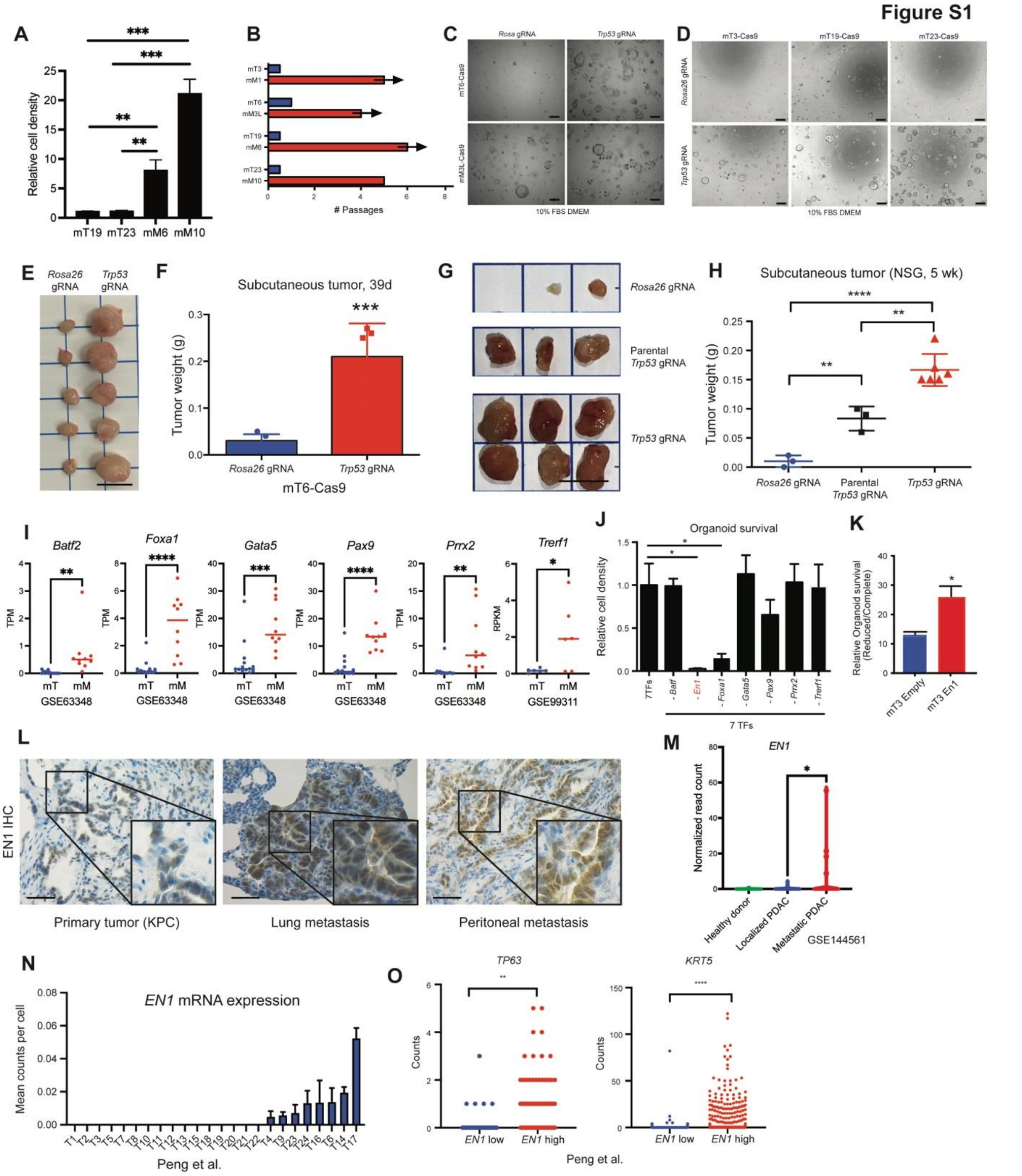

Figure S1. Identification of EN1 in organoid survival assay and its association with the PDA aggressive phenotype.

(A) Image-based quantification of mT and paired mM organoid survival in the reduced media 4 days post-cell seeding. n=3, mean ± SEM.

(B) Bar plot representing the number of passages the organoids underwent. Arrow indicating the organoids can be passaged continuously in the reduced media.

(C-D) Image representation of the development of organoid survival assay in the indicated mT and mM organoid pairs with *Trp53* knockout and *Rosa26* knockout control in the reduced media. Scale bar, 1mm

(E-F) Subcutaneous transplantation of mT6 organoids with *Trp53* knockout using CRISPR/Cas9. gRNA against *Rosa26* locus was used as a control. The tumors were imaged (E) and quantified for tumor weight (F) at 39 days post-injection. n=5 per group, mean ± SD. Scale bar, 10mm.

(G-H) After two gRNAs targeting wild-type *Trp53* were introduced in mT organoids (parental organoids), mT organoids were subjected to the reduced media in order to enrich p53 LOH mT organoids. These organoids (n = 3 for *Rosa26* gRNA, n = 3 for parental organoids and n = 6 for the enriched population for *p53* LOH) were subcutaneously transplanted in NSG mice. The tumors were collected 5 weeks post-injection (G) and quantified by tumor weight (I). Scale bar, 10 mm.

(I) *Batf2*, *Foxa1*, *Gata5*, *Prrx2*, *Pax9*, and *Trerf1* mRNA expression in mM organoids relative to mT organoids from Oni et al. (GSE63348) and Roe et al. (GSE99311). Each dot represents an organoid line.

(J-K) Relative cell density in the organoid survival assay from Figure 1C (J) and Figure 1D (K) was quantified. Mean ± SEM is shown.

(L) EN1 IHC of the pancreatic primary tumor, lung and peritoneal metastases from a KPC mouse. Scale bar, 100 µm.

(M) *EN1* normalized read count of primary purified circulating tumor cells from pancreatic cancer patients (localized and metastatic) and healthy donors from Franses et al. (GSE144561).

(N) *EN1* mRNA mean counts per cell of 24 pancreatic patients from Peng et al. scRNA-seq (CRA001160). Mean ± SD is shown.

(O) Squamous subtype markers, *TP63* and *KRT5* mRNA counts in *EN1*-high (T6, T14, T16, T17) vs. -low (T1, T2, T3, T5) PDA patients from Peng et al. scRNA-seq (CRA001160).

Unless otherwise indicated, *p*-values were determined by unpaired student's *t* test (two-tail) and *, **, ***, **** indicate *p*-val < 0.05, < 0.01, <0.001, <0.0001, respectively.

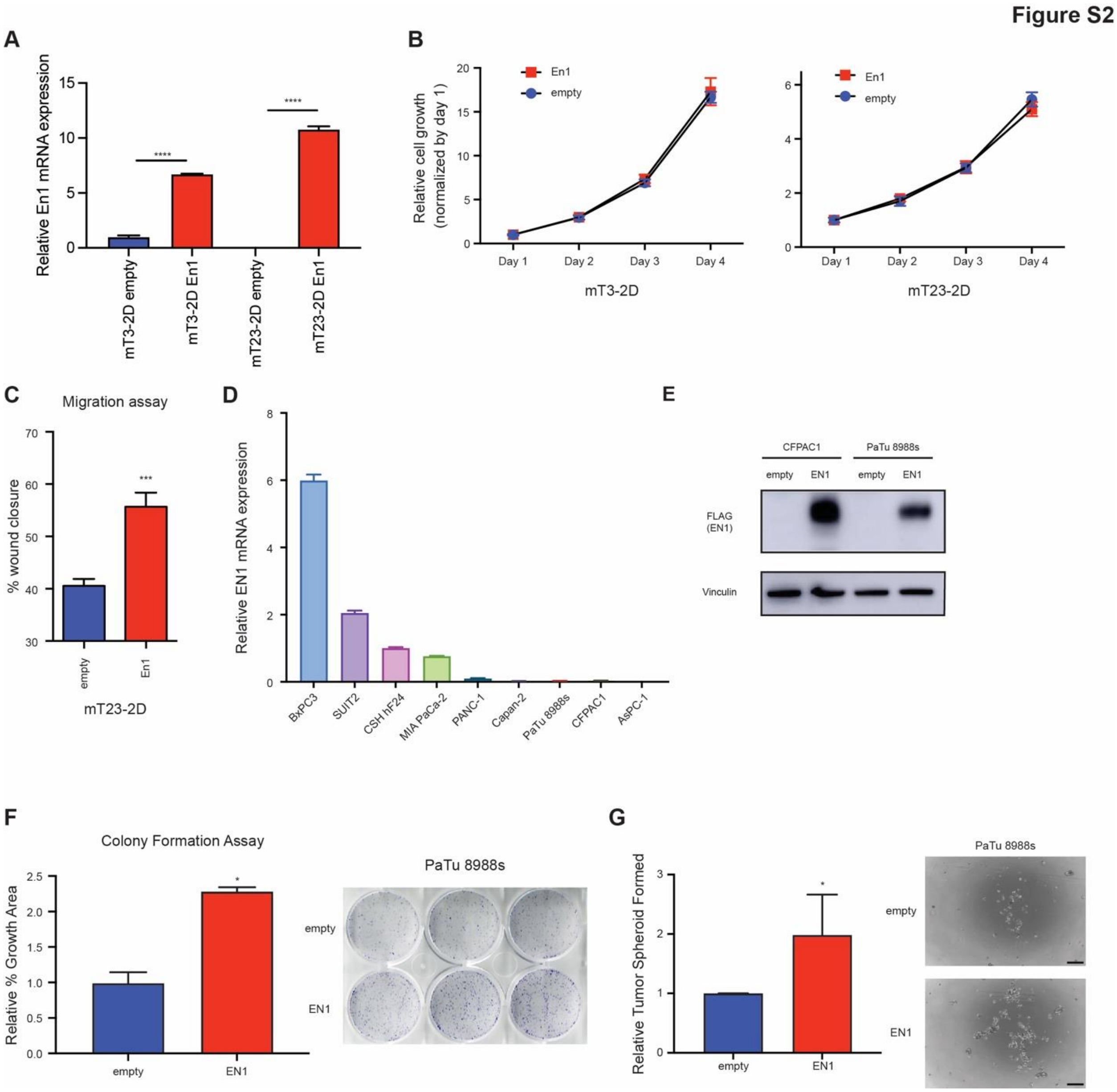

Figure S2. Gain-of-function experiments revealed that EN1 promotes PDA metastatic properties.

(A) Relative *En1* mRNA expression determined by RT-qPCR in mT3-2D and mT23-2D cell lines with (*En1*) and without (empty) *En1* cDNA overexpression.

(B) mT3-2D and mT23-2D cells with *En1* cDNA were subjected to cell proliferation assay compared to empty vector control. Cell proliferation rate was determined by ATP-based cell viability assay using CellTiterGlo and luminescence was measured daily for 4 days and normalized to day 1. n=3 per time point, mean ± SD.

(C) mT23-2D with *En1* cDNA were subjected to wound-healing assay compared to empty vector control, and the area of the closed wound was quantified at 0- and 24-hour post-scratching, and the percentage of wound closure was calculated. n=3, mean ± SD.

(D) Relative *EN1* mRNA expression in human PDA cell lines determined by RT-qPCR. n=3, mean ± SD.

(E) Western blot analysis to determine the protein expression of FLAG-tagged EN1 compared to the plasmid without *EN1* cDNA (empty) control in CFPAC1 and PaTu 8988s cell lines.
(F) PaTu8988s empty and *EN1* cells were subjected for colony formation assay for 14 days, and the colonies were stained by crystal violet (right) and quantified (left) by percentage growth area. n=3, mean ± SD.
(G) PaTu8988s empty and *EN1* cells were subjected for anchorage-independent tumor spheroid formation assay for 7 days, and the numbers of spheroids were monitored (right) and quantified (left). n=3, mean ± SEM. Scale bars, 350 μm.
Unless otherwise indicated, *p*-values were determined by unpaired student's *t* test (two-tail), and * and *** indicate *p*-val < 0.05 and <0.001, respectively.

Figure S3

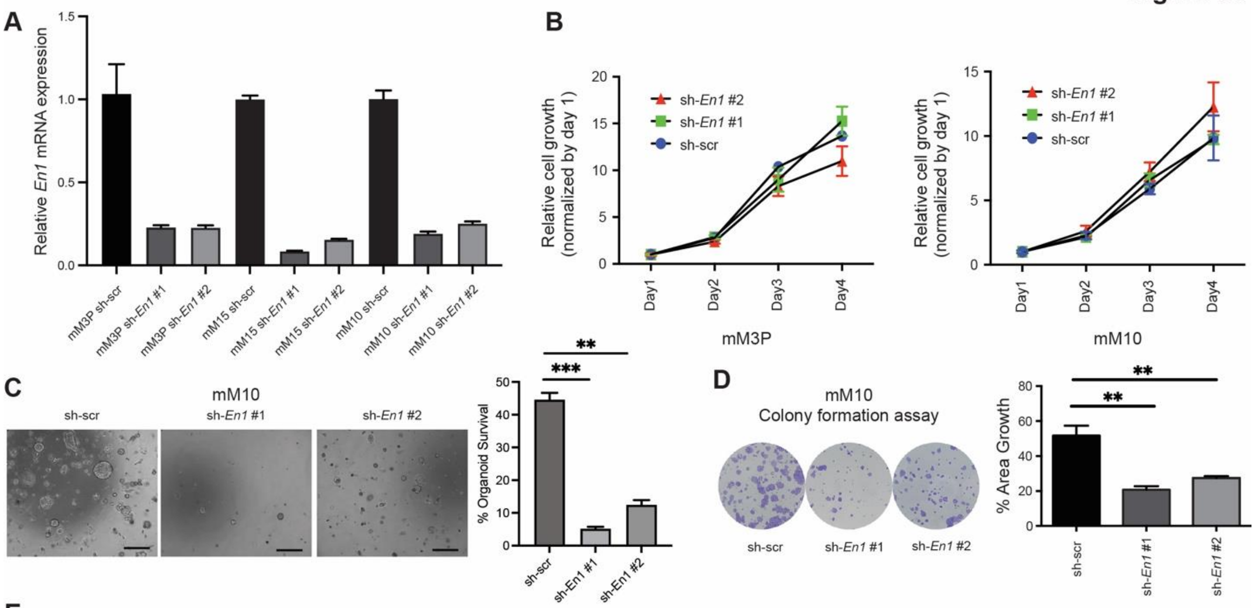

E

Case	Metastases from M3P/sh-scr orthotopic transplants #1	#2	#3	#4	#5	Metastases from M3P/shEn1 orthotopic transplants #1	#2	#3	#4
Liver	Met	Met	Met	Met	Met	No	No	Met	No
Lung	Micromets (>10)	Micromets (>10)	Micromets (>10)	Micromets (>10)	Micromets (<10)	Micromets (<10)	Micromets (>10)	Micromets (<10)	Micromets (<10)
Diaphragm	Met	Met	Met	Met	No	No	Met	Met	No
Ovary	Met	No	No	No	No	No	No	No	No
Mesentery	Met	Met	Met	No	No	No	Met	Met	No
Stomach	Met	No	No	No	No	No	No	No	No
Peritoneum	No	No	No	No	Met	No	Met	Met	No

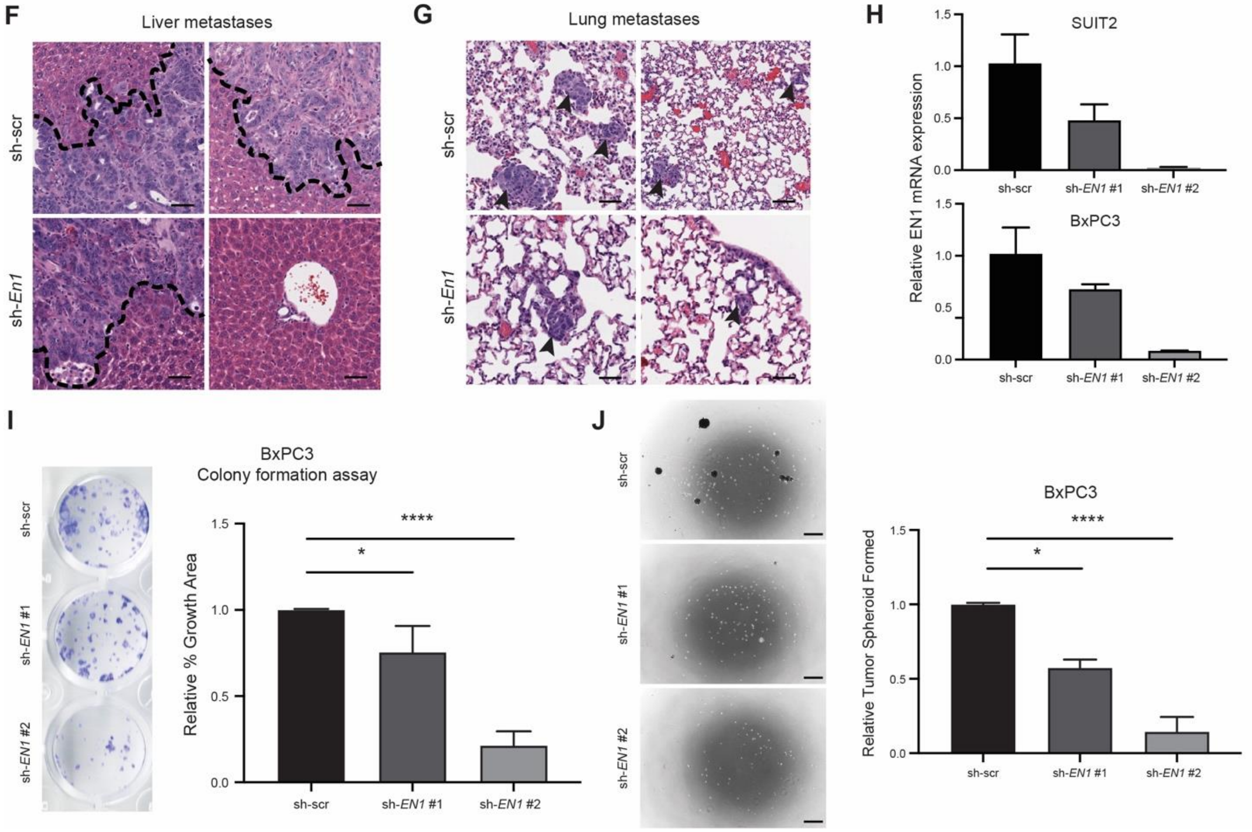

Figure S3. EN1 is necessary to acquire the metastatic characteristics of PDA.

(A) Relative *En1* mRNA expressions determined by RT-qPCR in mM3P, mM15, and mM10 organoids with shRNA targeting *En1* mRNA coding region (sh*En1* #1 CDS) and *En1* mRNA 3' untranslated region (sh*En1* #2 3'UTR) compared to the scramble shRNA (sh-scr) control organoid. n=3, mean ± SD.
(B) Image-based quantification of organoid growth for mM3P and mM10 shScr, sh*En1* #1, and sh*En1* #2 organoids in the complete media. Organoid growth was normalized to day 1. n=3, mean ± SD.
(C) shScr and sh*En1* mM10 organoids were subjected to organoid survival assay for 4 days (top) and quantification of organoids (bottom). Scale bars, 1mm.
(D) shScr and sh*En1* mM10 organoids were subjected to colony formation assay for 7 days, and the colonies were stained by crystal violet (left) and quantified (right) by percentage growth area.
(E) Summary of metastases from mM3P shScr (n=5) and sh-*En1* orthotopic transplants (n=5), 7 weeks post-transplantation. Depletion of En1 reduced liver metastasis frequency. Fisher's exact test, *p*-val <0.05.
(F) Representative H&E staining of liver metastasis from the orthotopic injections of mM3P shScr and sh*En1* organoids. Scale bar, 50 µm.
(G) Representative H&E staining of lung metastasis from the orthotopic injections of mM3P shScr and sh*En1* organoids. Scale bar, 50 µm.
(H) Relative EN1 mRNA expressions determine by RT-qPCR in SUIT2 (left) and BxPC3 (right) cell lines with shRNAs targeting *EN1* mRNA compared to the scramble shRNA. n=3, mean ± SD.
(I) shScr and sh*EN1* BxPC3 cells were subjected to colony formation assay for 2 weeks, and the colonies were stained with crystal violet (top) and quantified (bottom) for the percentage growth area. n=3, mean ± SD.
(J) shScr and sh*EN1* BxPC3 cells were subjected to anchorage-independent tumor spheroid formation assay for 7 days, and the numbers of spheroids were monitored (left) and quantified (right). n=3, mean ± SD. Scale bars, 350 µm.
Unless otherwise indicated, *p*-values were determined by unpaired student's *t* test (two-tail) and *, **, ***, **** indicate *p*-val < 0.05, < 0.01, <0.001, <0.0001, respectively.

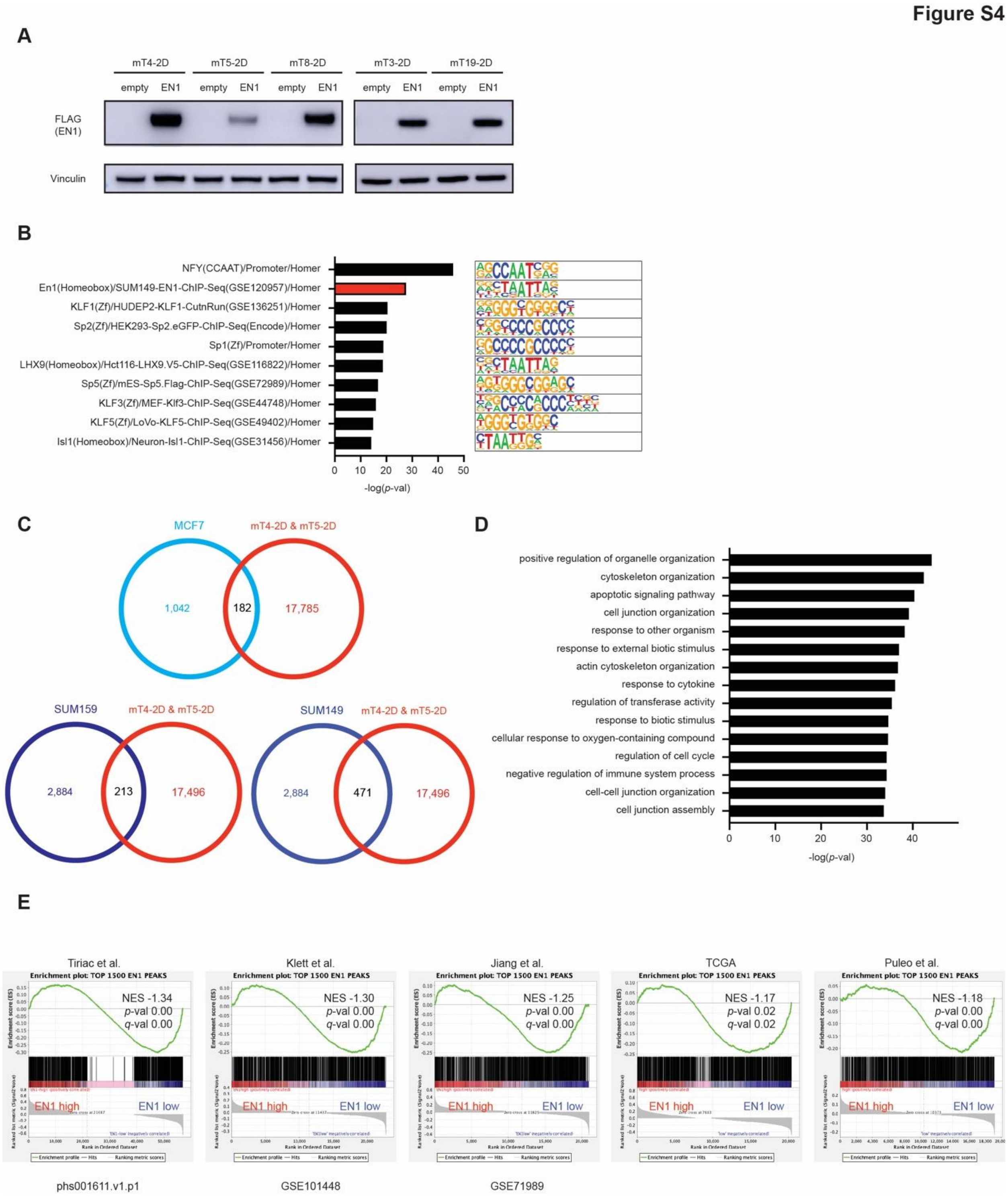

Figure S4. Characterization of EN1 binding regions in PDA genome.
(A) Western blot analysis to determine the protein expression of FLAG-tagged EN1 compared to the empty control in mT3-2D, mT4-2D, mT5-2D, mT8-2D, and mT19-2D cells.
(B) Homer motif analysis for the known motifs using the overlapping mT4-2D and mT5-2D EN1 peaks. *p*-value was determined by HOMER.

(D) Genomic Regions Enrichment of Annotations Tool (GREAT) analysis of the overlapping mT4-2D and mT5-2D EN1 peaks showing the top 15 enriched pathways in biological functions. *p*-value was determined by GREAT.

(C) Overlapping EN1 peaks identified in triple-negative breast cancer (TNBC) cells (MCF7, SUM149, and SUM159) and mT4-2D & mT5-2D cells. To properly compare murine and human genomes, we convert the EN1 peak positions identified in mT-2D cell lines (NCBI37/mm9) to human genome assembly (GRCh37/hg19) using UCSC Lift Genome Annotations tool. Of the 20,271 EN1 peaks identified in mT-2D cell lines, 17,967 peaks were LiftOver successfully. Among the TNBC cell lines, 182, 213, and 471 peak overlaps were identified in MCF7, SUM159, and SUM149 cells, respectively.

(E) GSEA of the top 1500 EN1 peak-associated genes in *EN1*-high vs. -low pancreatic cancer patients, organoids, or cell lines from Tiriac et al. (phs001611.v1.p1), Klett et al. (GSE101448), Jiang et al. (GSE71989), TCGA-PAAD, and Puleo et al. NES, *p*-value, and FDR *q*-value were determined by GSEA.

Figure S5

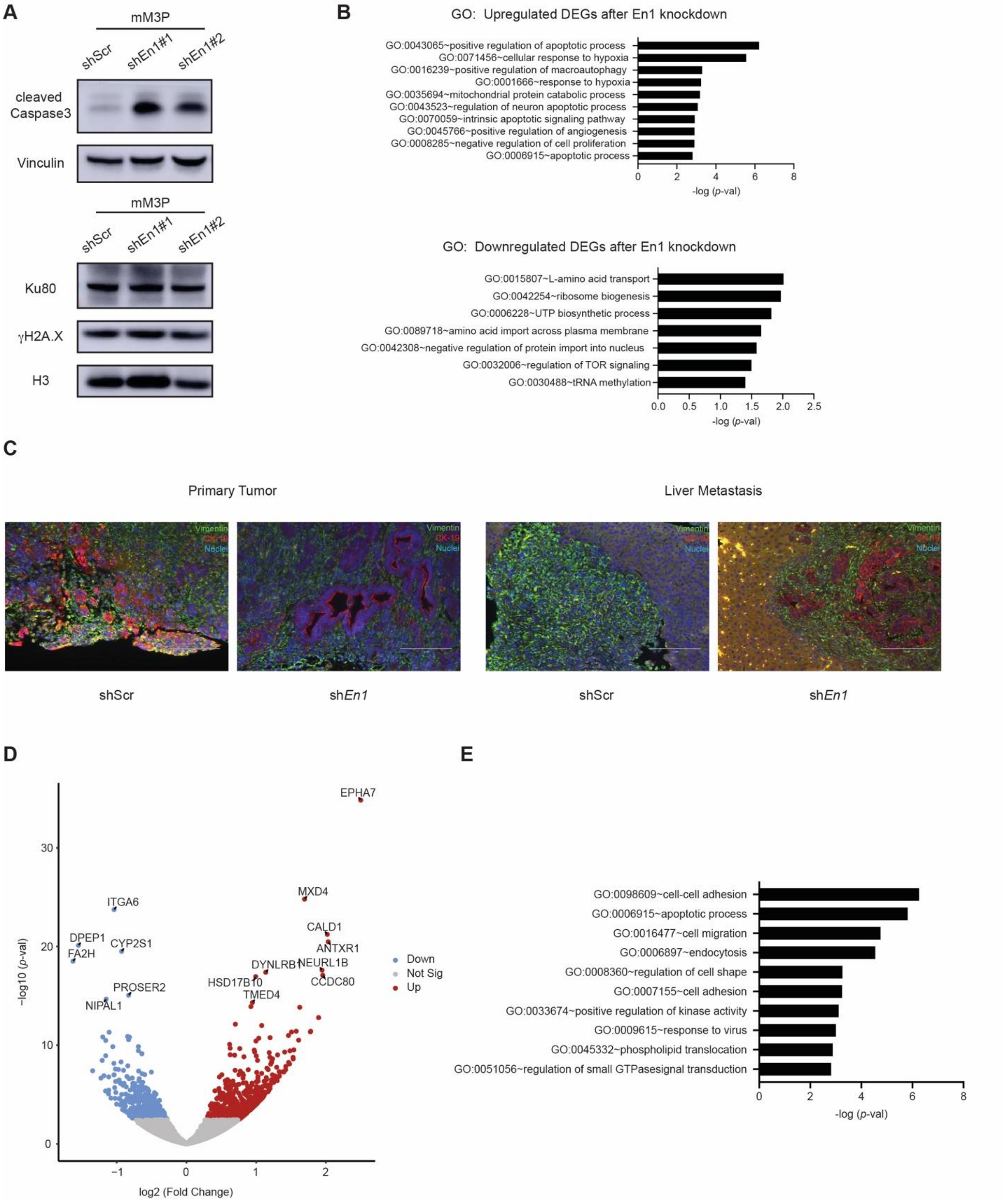

Figure S5. EN1 genomic targets were up-regulated upon EN1 depletion.
(A) Western blot analysis to determine the protein expression of cleaved Caspase 3, KU80 and γH2A.X. in mM3P organoids with scramble (shScr) and two independent *En1* (sh*En1*) shRNA constructs cultured in the reduced media for 24 hours before harvesting.

(B) DAVID analysis of the upregulated DEGs (top) and downregulated DEGs (bottom) after *En1* knockdown in mM3P and mM15 organoids showing the top enriched pathways in biology functions. *p*-value was determined by DAVID.
(C) Immunofluorescence staining of vimentin and CK-19 in the tissue sections collected from orthotopically transplanted mM3P organoids (figure 3D-F), comparing shScr and sh*En1* conditions, depicting primary tumor (left) and distant liver metastasis (right). Scale bar, 150 μm.
(D) Volcano plot representing RNA-sequencing of SUIT2 shScr and two independent sh*EN1* constructs identified 1056 differentially expressed genes (DEG). Among the DEGs, 638 genes were upregulated, and 418 genes were downregulated after *En1* knockdown.
(E) DAVID analysis of the DEGs after *EN1* knockdown in SUIT2 cells showing the top 10 enriched pathways in biology functions. *p*-value was determined by DAVID.

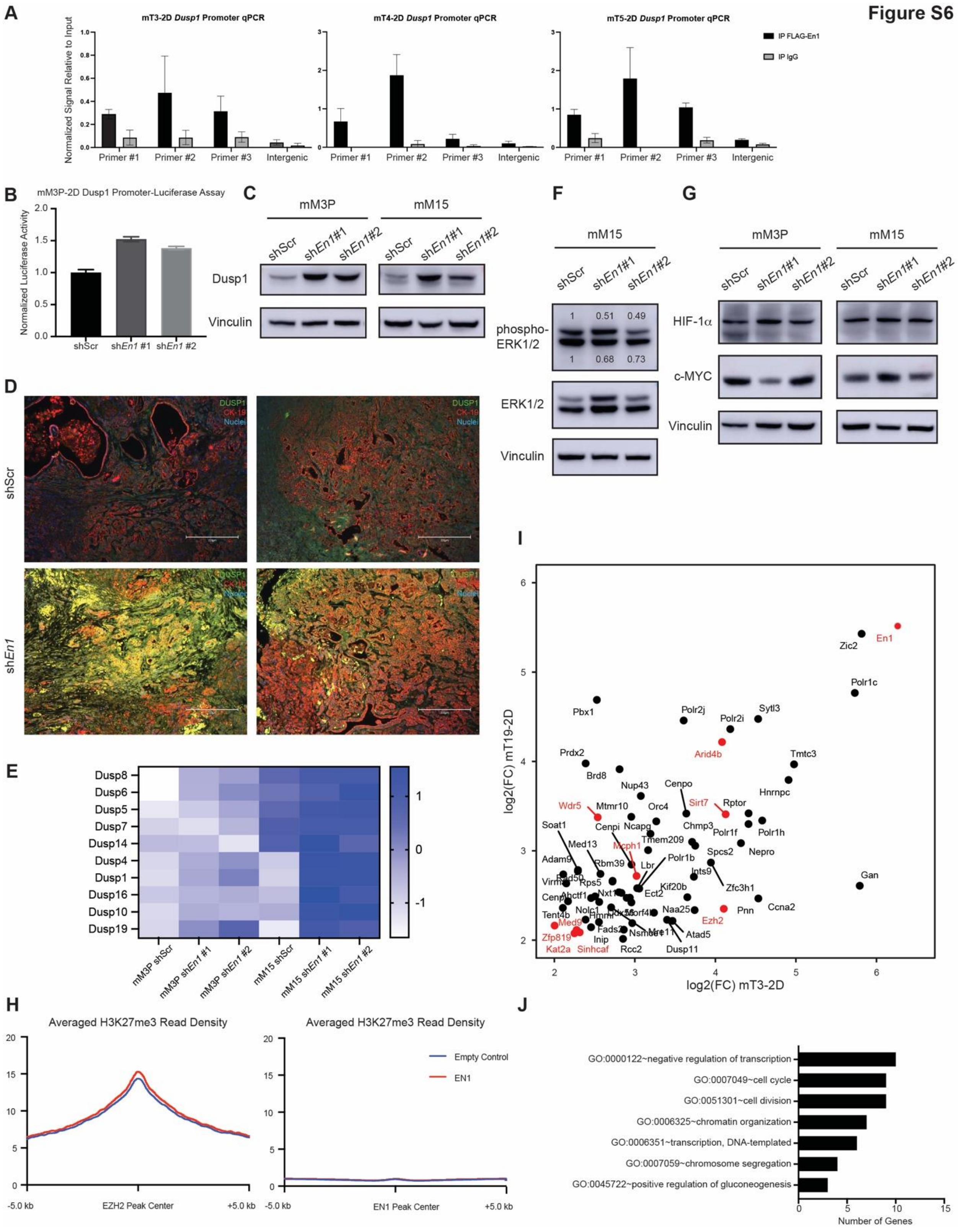

Figure S6. EN1 regulates MAPK pathways but not MYC or HIF-1α-dependent hypoxia responses and EN1-mediated transcription repression is independent of EZH2.

(A) CUT&RUN assay targeting FLAG-tagged EN1 in three mT-2D cell lines, followed by qPCR analysis employing three primer pairs spanning *Dusp1* promoter EN1 binding sites identified in figure 6E.
(B) Luciferase activity assay assessing the *Dusp1* promoter activity in mM3P-2D cell line with scramble (shScr) and two independent *En1* (sh*En1*) shRNA constructs (n=3).
(C) Western blot analysis to determine the protein expression of DUSP1 in mM3P and mM15 organoids with shScr and two independent sh*En1* constructs.
(D) Immunofluorescence staining of DUSP1 and CK-19 in primary tumor tissue sections collected from shScr (top) and sh*En1* (bottom) mM3P orthotopically transplanted organoids. Scale bar, 150 μm.
(E) Heatmap showing the expressions of *Dusp* genes in shScr and sh*En1* mM3P and mM15 organoids, as derived from the RNA-seq dataset presented in figure 5A.
(F) Western blot analysis to determine the protein expression of phospho-ERK1/2 (Thr202/Tyr204) and total ERK1/2 in mM15 organoids cultured in the complete media with scramble (shScr) and two independent *En1* (sh*En1*) shRNA constructs.
(G) Western blot analysis to determine the protein expression of HIF-1α and c-MYC in mM3P and mM15 organoids cultured in the reduced media for 24 hours with scramble (shScr) and two independent *En1* (sh*En1*) shRNA constructs. Vinculin data for mM3P was duplicated as Figure 6F right panel.
(H) Averaged density plots of H3K27me3 CUT&RUN-seq signals around EZH2 genomic binding sites (left) and EN1 genomic binding sites (right) in mT4-2D, mT5-2D, and mT8-2D empty | *FLAG-EN1* cells.
(I) Nuclear co-immunoprecipitation assay of FLAG-tagged EN1 followed by mass spectrometry in mT3-2D and mT19-2D EN1 overexpressed cell lines identified 68 EN1-interacting proteins. Proteins involved in transcription repression were highlighted in red.
(J) DAVID analysis of the EN1 interacting proteins showing the top significantly enriched pathways in biological functions.

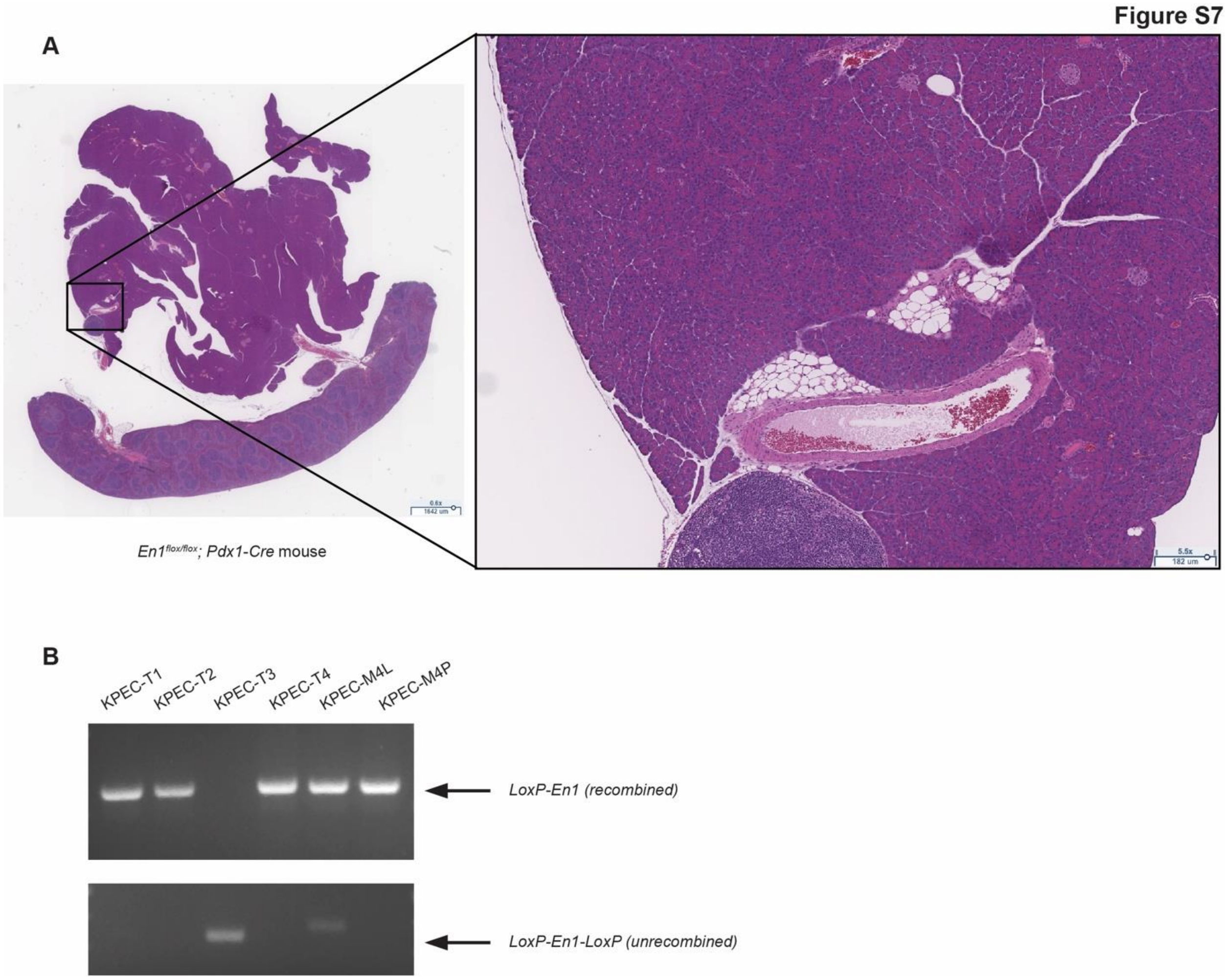

Figure S7. *En1* knockout does not affect the development of murine pancreas and Cre recombination is not prevalent in all animals.
(A) H&E staining of pancreas isolated from *En1*$^{flox/flox}$*; Pdx1-Cre* (EC) mouse at 67 days age.
(B) PCR analysis of 1 loxP-*En1* (recombined, top) and LSL-*En1* cassette (unrecombined, bottom) using tumor and metastasis organoids derived from KPEC mice. T: tumor; M: metastasis; L: liver; and P: peritoneum. Expected En1 1 loxP size: 600 bp; expected LSL-En1 size: 380 bp.

Table S1. Nuclear co-immunoprecipitation of FLAG-tagged EN1 followed by mass spectrometry.
Table S2. Oligonucleotide list.
Table S3. Antibody list.
